# Sensitivity Analysis of Process Parameters on Deposition Quality and Multi-Objective Prediction in Ion-Assisted Electron Beam Evaporation of Ta_2_O_5_ Films

**DOI:** 10.3390/mi17020166

**Published:** 2026-01-27

**Authors:** Yaowei Wei, Jianchong Li, Wenze Ma, Hongqin Lei, Fei Zhang, Zhenfei Luo, Henan Liu, Xianghui Huang, Linjie Zhao, Mingjun Chen

**Affiliations:** 1 School of Mechanical and Power Engineering, Shanghai Jiao Tong University, Shanghai 200240, China; 2 Laser Fusion Research Center, China Academy of Engineering Physics, Mianyang 621900, China; 3 School of Mechatronics Engineering, Harbin Institute of Technology, Harbin 150001, China

**Keywords:** Ta_2_O_5_ film, ion-assisted electron beam evaporation, refractive index, surface roughness, physics-informed Bayesian optimization

## Abstract

Tantalum pentoxide (Ta_2_O_5_) films deposited on fused silica substrates are critical components of high-power laser systems. Ion-assisted electron beam evaporation (IAD-EBE) is the mainstream technique for fabricating Ta_2_O_5_ films. However, it commonly requires extensive experimental efforts for deposition quality optimization, while each coating cycle is extremely time-consuming. To solve this issue, this work establishes a dataset targeting the surface roughness (*Rq*) and refractive index (*n*) of Ta_2_O_5_ films using atomic force microscopy, as well as ellipsometer and deposition experiments. Influence of assisting ion source beam voltage (*V*)/current (*I*) and Ar (*Q*_1_)/O_2_ (*Q*_2_) flow rate on the *n* and *Rq* of Ta_2_O_5_ films are analyzed. Combining energy-field mechanism analysis with a Bayesian optimization approach (PI-BO), both deposition quality prediction and feature analysis of process parameters are achieved. The determination coefficient/mean absolute error for the prediction models of *n* and *Rq* reach 0.927/0.013 nm and 0.821/0.049 nm, respectively. Based on sensitivity analysis, the weight factors of *V*, *I*, *Q*_1_, and *Q*_2_ affecting *n*/*Rq* of Ta_2_O_5_ films are determined to be 0.616/0.274, 0.199/0.144, 0.113/0.582, and 0.072/0.000. *V* and *Q*_2_ are identified as the core factors for regulating deposition quality. The optimal ranges for *V* and *Q*_2_ are 600~700 V and 70~80 sccm, respectively. This study proposes a PI-BO method for predicting *Rq* and *n* of Ta_2_O_5_ films under small-data conditions, while determining the preferred parameter ranges and their sensitivity weight factors. These findings provide effective theoretical support and technical guidance for IAD-EBE strategy design and optimization of optical films in high-power laser systems.

## 1. Introduction

Tantalum pentoxide (Ta_2_O_5_) has emerged as a core material in high-energy laser systems due to its high refractive index (*n*), low absorption loss, and excellent chemical stability [1,2,3,4,5,6]. Ion-assisted electron beam evaporation (IAD-EBE), a pivotal physical vapor deposition technique, allows precise control of density and stoichiometry of Ta_2_O_5_ films through ion bombardment [7,8,9,10]. During IAD, an ion source supplies energetic particles that enhance adatom mobility, reduce defect state density, and promote the formation of a dense microstructure. This process thereby facilitates the deposition of high-quality Ta_2_O_5_ films [11,12,13,14]. However, the superiority of this process heavily relies on the synergistic regulation of multiple parameters, which exhibit complex nonlinear coupling effects. This complexity poses significant challenges, such as high costs and poor interpretability, when attempting to identify the optimal deposition quality through traditional experimental approaches [15,16,17,18].

Current research on IAD processes predominantly focuses on the isolated impact of individual parameters on the deposition quality [19]. For instance, Mansour et al. [12] investigated the influence of ion energy and current density on the *n*, densification, and extinction coefficient of Ta_2_O_5_ films deposited via IAD. They reported maximum *n* of 2.27 at the ion energy of 300 eV and at the ion current density of 60 μA/cm^2^. Chun et al. [20] systematically established a quantitative relationship between assisting ion source beam voltage (*V*) and film performance in IAD deposition by controlling this single variable. They examined the effect of Ar/O_2_ plasma post-treatment duration on multilayer film properties. Demiryont et al. [21] studied oxygen content variation in ion beam sputtered Ta_2_O_5_ films. They revealed that an optimized Ar/O_2_ ratio yielded a bandgap of 4.3 eV and low extinction in the visible range. Sakiew et al. [22] found that xenon increased deposition rates but carried the risk of inducing localized defects at high currents. Traditional experimental methods, which rely solely on trial-and-error or univariate analysis, are inadequate for accurately analyzing such high-dimensional nonlinear relationships. Moreover, they are unable to deeply explore or reliably predict the complex relationship from small-sample, high-cost experimental datasets.

In recent years, machine learning (ML) has provided robust support for the inverse design of materials and processes. It demonstrates significant advantages in predicting optical responses of known materials and optimizing multilayer film architectures [23,24,25,26]. Tran et al. [27] developed a toolkit integrating electromagnetic simulation with ML to efficiently design multilayer optical films. This toolkit enabled rapid and accurate prediction of optical performance from film structures. Harsh et al. [28] proposed a ML-based approach to automatically extract *n* from charts and construct predictive models. By employing XGBoost algorithms and incorporating features (material composition, synthesis parameters, etc.), they achieved high-precision prediction of *n*. Fan et al. [29] introduced a novel method termed thin-film neural networks (TFNNs), which exploits the structural similarity between multilayer films and neural networks. When handling a 232-layer film, TFNNs reduced the iteration time from 67.5 s with conventional simulation to 0.9 s. They successfully designed filter films that mimic the spectral response of human cone cells. Jiang et al. [30] proposed a Deep Q-learning-based optimization method for multilayer optical film design. In this method, reinforcement learning autonomously adjusted layer thicknesses to minimize the discrepancy between simulated and target spectra, as validated through applications such as solar absorbers. However, directly transferring these methods to parameter-performance modeling for IAD-EBE-based Ta_2_O_5_ film faces two fundamental challenges. The first is the data challenge: acquiring sufficient, balanced, high-quality experimental data for complex processes such as IAD is prohibitively expensive, resulting in scarce and unevenly distributed samples. The second is the model challenge: under small-sample conditions, data-driven deep neural networks are prone to overfitting. Consequently, their predictions often lack both extrapolation robustness and physical interpretability, offering limited guidance for practical process optimization. Therefore, developing an intelligent modeling approach that can effectively integrate process physics and achieve high predictive accuracy is essential. Such an approach must also maintain strong generalization capability under small-sample constraints, which is key to overcoming the optimization bottleneck in IAD processes.

In this work, the effect of assisting ion source beam current (*I*), *V*, and gas flow rate on the *n* and surface roughness (*Rq*) of Ta_2_O_5_ films is investigated. To solve the difficulties of multivariate nonlinear coupling and small-sample modeling, a physics-informed Bayesian optimization (PI-BO) method is proposed, which incorporates deposition mechanism as constraints. Meanwhile, the critical role of ion source parameters is quantitatively clarified, and its optimal ranges are identified. This study provides a significant theory basis and optimization methodology for preparing high-performance Ta_2_O_5_ films via IAD-EBE.

## 2. Experimental Details and Theoretical Method

### 2.1. Ta_2_O_5_ Films on the FSS Prepared by IAD-EBE Experiments

**Figure 1:**
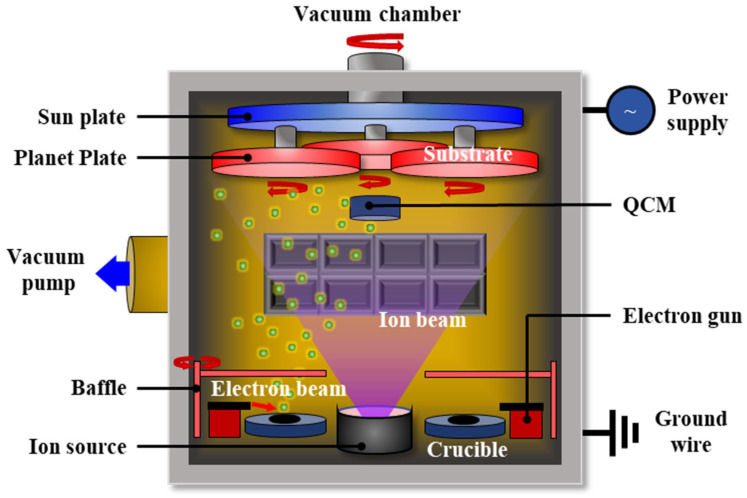
The schematic of ion-assisted electron beam evaporation fabrication process of Ta_2_O_5_ films on the fused silica substrates.

Ta_2_O_5_ films were deposited on fused silica substrates (FSS) using an IAD-EBE coating system (Chengdu, China). High-purity Ta_2_O_5_ particles (≥99.99%, 4 N) were used as the evaporation material after vacuum drying. The substrates were first wiped with dust free paper soaked in anhydrous ethanol to remove visible contaminants. They were then ultrasonically cleaned in acetone, ethanol, and deionized water for 15 min in sequence, and finally vacuum-dried before deposition. The coating system was equipped with a radio frequency (RF) discharge ion source. It provided an auxiliary ion beam with high energy density, excellent beam uniformity, and continuously adjustable parameters. Its primary role was to optimize film performance via high-energy ion beams. It densified the film microstructure, suppressed pore defects, and activated the FSS to enhance the film–substrate adhesion. A high-energy electron beam was used to bombard the Ta_2_O_5_ target, causing its evaporation and subsequent deposition onto substrates under planetary rotation. This process produced high-quality optical films, as illustrated in Figure 1.

Before deposition, the chamber was evacuated by a mechanical pump and a cryogenic pump to a base pressure of 3.0 × 10^−4^ Pa. During deposition, Ar and O_2_ were introduced through mass-flow controllers, and the chamber working pressure was continuously monitored by a vacuum gauge. A closed-loop pressure-control scheme was employed: the pumping conductance was dynamically adjusted based on the gauge feedback to maintain the chamber pressure at the target setpoint. The substrate temperature was actively controlled and maintained at 100 °C throughout the deposition process using the built-in substrate heating system (feedback control), and it was monitored in real time to minimize thermal fluctuations and eliminate temperature-induced variability. The film thickness was fixed at 400 nm and the deposition rate at 0.8 Å/s. A quartz crystal microbalance (QCM) was used for real-time monitoring, ensuring that variations in surface quality were primarily governed by the deposition conditions. *V*, *I*, and argon (*Q*_1_)/oxygen (*Q*_2_) flow rate were varied to study their effects on film surface quality. The detailed adjustment ranges of these deposition parameters are listed in Table 1. The parameter ranges were determined based on a combination of equipment operational limits, process stability considerations, and preliminary screening experiments. Specifically, the selected ranges ensured stable evaporation of Ta_2_O_5_, reliable ion source discharge without abnormal arcing, and repeatable film growth. In addition, the ranges were chosen to sufficiently cover regimes where ion-assisted densification, resputtering, and oxidation-state transitions are expected to occur.

### 2.2. Characterization of Rq and n in Ta_2_O_5_ Films

The diameter and thickness of the experimental FSS used for depositing Ta_2_O_5_ films were 10 mm and 1 mm, respectively. A comprehensive suite of techniques was employed to characterize the morphology, composition, and structure of the deposited films.

**Figure 2:**
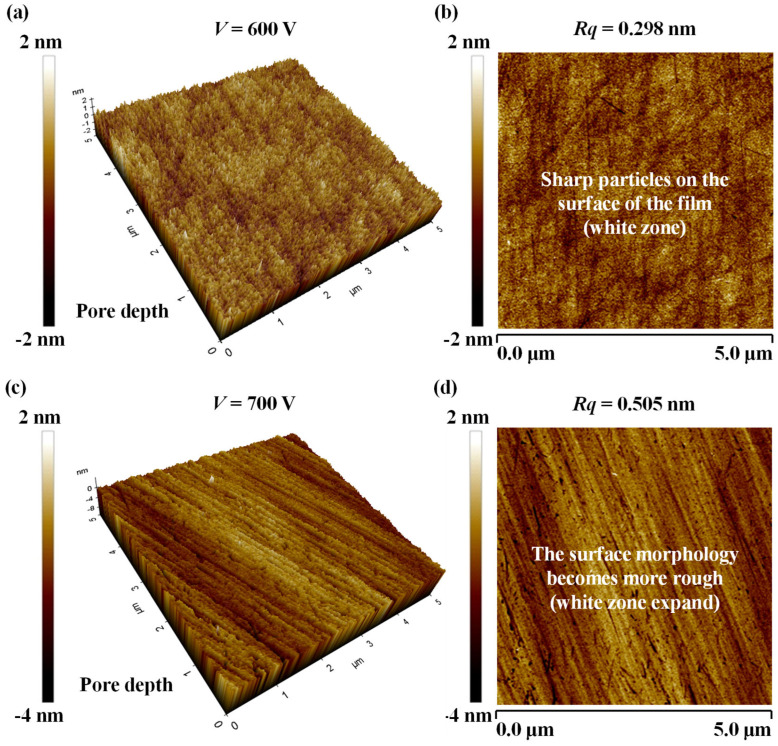
AFM surface morphology images of Ta_2_O_5_ film evaporated by ion-assisted electron beam evaporation under different process parameters. (**a**) Three-dimensional AFM topography (*V* = 600 V, *I* = 1200 mA, *Q*_1_ = 20 sccm, *Q*_2_ = 100 sccm). (**b**) Two-dimensional AFM surface morphology at 600 V (*Rq* = 0.298 nm). (**c**) three-dimensional AFM topography (*V* = 700 V, *I* = 1200 mA, *Q*_1_ = 20 sccm, *Q*_2_ = 100 sccm). (**d**) Two-dimensional AFM surface morphology at 700 V (*Rq* = 0.505 nm).

The *Rq* of the Ta_2_O_5_ films was characterized using atomic force microscopy (AFM) in tapping mode to minimize damage to the film surface (Chengdu, China). The instrument was regularly calibrated: the XY scan size with a standard grating and the *Z*-axis height with a step sample. The scanning range was set to 5 × 5 μm^2^ with a resolution of 512 × 512 pixels. After acquiring the raw topography images, plane-flattening was applied to remove background tilt. *Rq* was calculated as the root mean square deviation of the surface height, providing a sensitive measure of surface quality suitable for optical films. Figure 2 presents the three-dimensional (Figure 2a,c) and corresponding two-dimensional (Figure 2b,d) AFM topographies for films deposited at *V* of 600 V and 700 V, respectively, with other parameters held constant (*I* = 1200 mA, *Q*_1_ = 20 sccm, *Q*_2_ = 100 sccm). The film deposited at 600 V exhibits an exceptionally smooth and homogeneous surface with an *Rq* of 0.298 nm (Figure 2a,b). In stark contrast, increasing the ion beam voltage to 700 V significantly altered the surface topography, resulting in a more textured and granular morphology with a substantially higher *Rq* of 0.505 nm (Figure 2c,d). This indicates that the higher ion energy promoted greater surface mobility of adatoms or induced mild sputtering effects, leading to the development of a more textured morphology.

**Figure 3:**
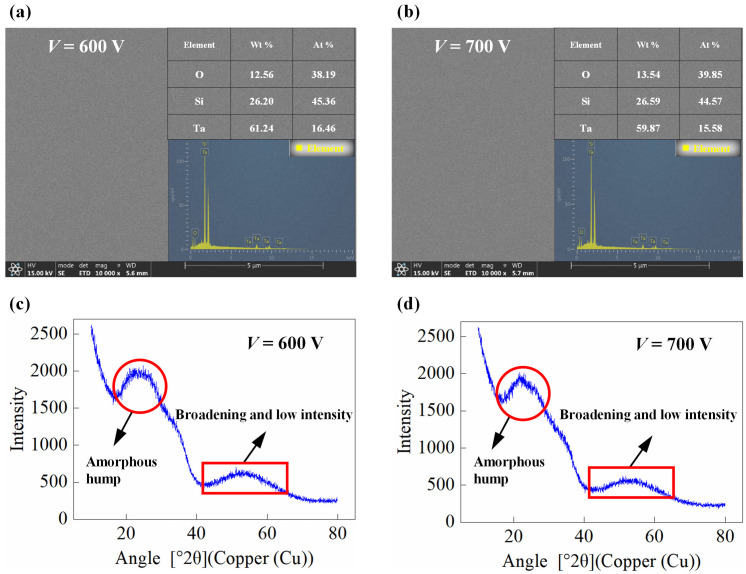
SEM, EDS, and XRD characterization results of Ta_2_O_5_ films under different assisting ion source beam voltage. (**a**) SEM surface image of the 600 V film (inset: corresponding EDS elemental composition results). (**b**) SEM surface image of the 700 V film (inset: corresponding EDS elemental composition results). (**c**) XRD pattern of the 600 V film (Cu Kα radiation, ω = 1°). (**d**) XRD pattern of the 700 V film (Cu Kα radiation, ω = 1°).

The elemental composition and surface morphology were analyzed using a scanning electron microscope (SEM) equipped with an energy-dispersive X-ray spectroscopy (EDS) detector (Chengdu, China). Prior to SEM/EDS analysis, the samples were coated with a thin conductive layer (Au) to prevent charging. The surface morphology was further corroborated by SEM, and the elemental composition was analyzed via EDS. Figure 3a,b show the SEM surface images for the 600 V and 700 V films, respectively. The SEM observations are fully consistent with the AFM results: the surface morphology of the film prepared under the 600 V process parameters is smoother. The corresponding quantitative EDS results are inset in each figure. Both films consist primarily of Ta and O, with a detectable Si signal originating from the underlying fused silica substrate, confirming the films are thinner than the SEM-EDS interaction volume. The atomic percentages of Ta are 16.46 at.% and 15.58 at.% for the 600 V and 700 V films, respectively (Figure 3a,b insets). The measured O/Ta atomic ratios for both films are notably lower than the stoichiometric value of 2.5 for Ta_2_O_5_. The substrate SiO_2_ provided the majority of the Si signal and a considerable portion of the O signal, while the film provided all the Ta signal and the remaining part of the O signal. Under the current deposition conditions, an oxygen-deficient tantalum oxide (TaO_x_) might have been formed.

The film structure was critically assessed by XRD (Chengdu, China) using Cu Kα radiation (λ = 1.5406 Å). The grazing Incidence XRD scan was performed continuously over 2θ = 10°~80° (step size: 0.035°, 2000 points) at a fixed grazing incidence angle (ω = 1°). The resulting patterns are shown in Figure 3c (600 V) and Figure 3d (700 V). The characteristic broad asymmetric diffraction hump spanning approximately 15° to 35° results from the superimposed scattering contributions of the amorphous FSS (dominant peak near 22°) and the amorphous Ta_2_O_5_ (α-Ta_2_O_5_) film, which typically exhibits a diffuse maximum at higher angles (approximately 28~35°) due to its distinct short-range atomic arrangement. Crucially, no sharp Bragg diffraction peaks corresponding to any known crystalline tantalum oxide polymorphs are observed. However, a very weak and broad intensity modulation is discernible around 50.5° in the patterns of both films, a position that coincides with the (221) reflection of crystalline β-Ta_2_O_5_. The extreme broadening and low intensity of these signals suggest that, if they originate from crystallized material, they correspond to extremely low-volume fraction within the dominant amorphous matrix. Nevertheless, the energy input under both conditions remained insufficient to drive a full-scale, long-range crystallization transition, which is consistent with the ion-assisted deposition process at relatively low substrate temperatures. This finding indicates that the deposition conditions, even at the higher ion energy of 700 V, did not provide sufficient energy or thermal activation for the formation of a long-range ordered crystalline lattice in these films.

Ellipsometry was used for high-precision and non-destructive determination of the n at 1053 nm (Chengdu, China). Before measurement, the samples were cleaned, dried, and inspected to select uniform surface regions. The instrument polarization state, wavelength, and angle of incidence were calibrated to minimize systematic errors. Measurements were then repeated at multiple sites under stable temperature and humidity.

### 2.3. Deposition Quality Prediction Achieved by Physics-Informed Bayesian Optimization

**Figure 4:**
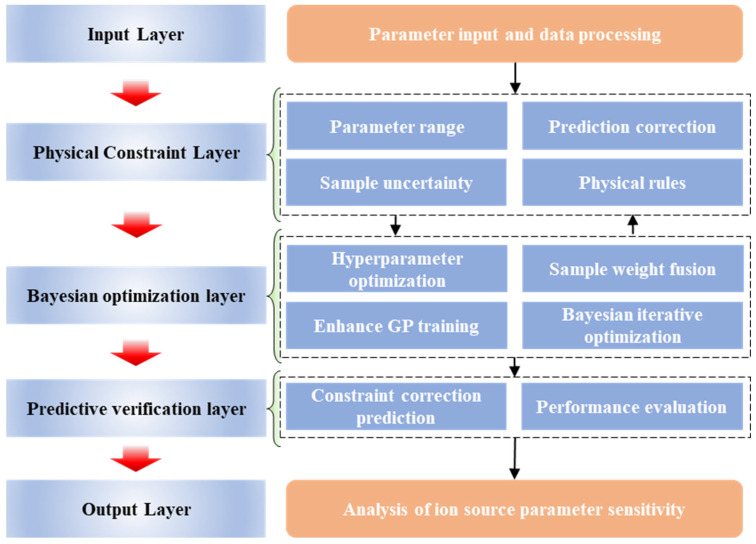
A complete hierarchical architecture of Bayesian optimization model integrating physical constraints for Ta_2_O_5_ film deposition process.

Figure 4 illustrated the complete hierarchical architecture of the proposed PI-BO model. Inputs to this architecture consisted of ion source parameters (*V*, *I*, *Q*_1_, and *Q*_2_), while outputs were *Rq* and *n* of Ta_2_O_5_ films. Overfitting and poor physical interpretability represented critical challenges in modeling small-sample process data. In this study, these were addressed through the deep coupling of the physical process prior of film deposition and data-driven BO. Preprocessing of raw data was performed in the input stage. Data standardization was implemented via the Z-score method within the parameter input and data processing module, followed by partitioning of training/test sets (8:2). Outlier filtering based on the interquartile range (IQR) criterion was also conducted, thereby providing structured and high-reliability foundational data for subsequent modeling steps.

The physical constraint layer was the main component responsible for ensuring model interpretability. It included parameter range constraints, prediction result correction constraints, sample uncertainty constraints, and physical prior rule constraints. The parameter range constraint limited the legal intervals between the input process parameters and the output *Rq*/*n*, as well as filtered out invalid samples. For the uncertainty constraints of data features, the threshold for repeated samples was set to 2, the basic uncertainty to 0.12, and the domain penalty coefficient to 0.5. These values were chosen to adapt to the stability characteristics of identical parameter combinations, the overall fluctuation range of the data, and the focus requirements of the low-*Rq* parameter domain, respectively, thereby enhancing model robustness. The prediction result correction constrained fine-tune the original predicted values of the GP model according to physical rules to ensure that the output conforms to the process laws. For the optimization goals of *Rq* and *n*, five parameter combination scoring rules were respectively constructed by physical prior constraints. The weight distribution was positively correlated with the influence intensity of each parameter on surface quality. The rule threshold was jointly determined by data characteristics and physical laws.

The BO layer employed the enhanced Gaussian Process (GP) as its core surrogate model to enable data-driven accurate modeling. First, sample weights output by the physical constraint layer were incorporated into the training process. Subsequently, iterative optimization of the GP model’s hyperparameters was accomplished via 5-fold cross-validation, where the optimal ARD squared exponential kernel function and noise parameter Σ were identified. Based on the Bayesian iterative optimization strategy, the model’s prediction error was minimized within the valid parameter space constrained by physical rules. This led to the construction of an enhanced GP model with both high fitting accuracy and strong generalization capability. In the prediction and validation phase, the raw GP outputs were further fused with physical prior rules through the constraint-corrected prediction module, and final *Rq* predictions that conform to process laws were generated. Meanwhile, model performance evaluation was conducted using quantitative metrics. In the output layer, the influence weights of each ion source process parameter on *Rq* were clarified via feature sensitivity analysis.

## 3. Results and Discussion

### 3.1. Mechanistic Analysis of the Influence of Plasma Energy Fields on Rq and n of Ta_2_O_5_ Films

**Figure 5:**
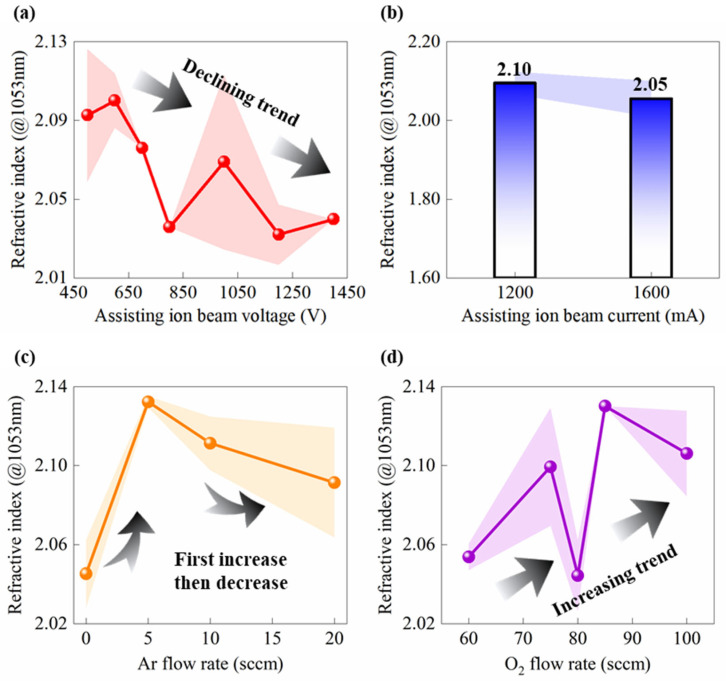
The influence of characteristic parameters on *n* (@1053 nm) of Ta_2_O_5_ optical films. (**a**) Assisting ion source beam voltage—*V* (500~1400 V). (**b**) Assisting ion source beam current—*I* (1200~1600 mA). (**c**) Ar flow rate—*Q*_1_ (0~20 sccm). (**d**) O_2_ flow rate—*Q*_2_ (60~100 sccm). Arrows indicate the general trend of the data; shaded bands represent uncertainty bands.

As crucial characteristics, *n* and *Rq* determine the service performance of Ta_2_O_5_ films in the high-energy laser systems. The role of ion source in affecting the deposition quality achieved by IAD-EBE is extremely important. This section investigates the effect of ion source parameters on *n* and *Rq*, as well as elucidating the underlying mechanisms governing these influences. Figure 5 illustrates the dependence of *n* (measured at 1053 nm) on four key ion source parameters: *V*, *I*, *Q*_1_, and *Q*_2_. Figure 5a reveals that *n* decreases overall with increasing *V*. *n* attains a local maximum at approximately 600 V. It drops sharply to around 2.03 at 800 V. A slight recovery in *n* is observed near 1000 V. It remains at a low level of 2.03~2.05 in this interval. Low *V* (500~650 V) favors the attainment of high *n*. However, a further rise in *V* induces an overall reduction in *n*. At low *V* (<650 V), ion bombardment during deposition regulates the columnar microstructure growth of films. This modification promotes film densification, thereby contributing to a higher value of *n* [12,31]. The decrease in *n* above 600 V may be attributed to the detrimental effect of excessive ion bombardment energy [12,32]. In Figure 5b, *n* exhibits an almost linear decrease as *I* increases from 1200 mA to 1600 mA. Its average value drops from 2.095 to 2.054, with error bar distribution further verifying the robustness of this trend. In principle, higher ion flux is typically anticipated to enhance the densification of deposited films [20]. Within the relatively high current range, the incident ion flux is significantly elevated. The resultant intense ion bombardment intensifies resputtering and facilitates the formation of local micropores or porous domains, which consequently lowers *n* [12,33]. Thus, an excessively high *I* is detrimental to the fabrication of Ta_2_O_5_ films with high *n*.

Figure 5c shows that *n* first increases and then decreases with increasing *Q*_1_. When *Q*_1_ increases from 0 sccm to 5 sccm, *n* rises sharply from about 2.05 to about 2.14, which is the highest value among all samples. As *Q*_1_ is further increased to 10 and 20 sccm, *n* gradually decreases and approaches about 2.09. At low *Q*_1_, the discharge is unstable and the ion beam flux is relatively low. The ion-assisted effect is therefore insufficient, and the film tends to grow with pronounced nanoscale voids, which results in a reduced refractive index. When a moderate amount of Ar is introduced, the discharge stability and ionization efficiency are significantly improved. More efficient momentum transfer to the film surface is achieved, the structure becomes more uniform and denser, and *n* reaches a maximum [31,34]. When *Q*_1_ is too high, Q_2_ becomes low and the supply of oxygen atoms is insufficient. During deposition, this favors the formation of sub-stoichiometric TaO_x_ (x < 2.5), whose *n* is generally lower than that of stoichiometric Ta_2_O_5_. In addition, a high *Q*_1_ may strengthens resputtering, which weakens densification and reduces *n* [35]. Figure 5d shows that, as *Q*_2_ increases from 60 sccm to 100 sccm, *n* exhibits an overall slow increase, with pronounced local fluctuations. In reactive ion-assisted deposition, O_2_ plays a crucial role in controlling the oxidation state and stoichiometry of the film. In general, an increase in *Q*_2_ is beneficial for improving the oxidation degree of the film, the thermal decomposition and oxygen loss of Ta_2_O_5_ under high temperature and ion bombardment are suppressed, and the concentration of oxygen vacancies is reduced. As a result, a gradual increase in n is observed for the deposited Ta_2_O_5_ films. It should be noted that partial oxygen loss can still occur despite using Ta_2_O_5_ as the evaporation source, even in the vapor phase and growing film under electron beam bombardment and vacuum transport. Sub-stoichiometric TaO_x_ (x < 2.5) can thus be formed, and the final stoichiometry becomes highly sensitive to the local *Q*_2_ and plasma chemistry near the substrate [36,37]. Near an *Q*_2_ of 75~80 sccm, n and its standard deviation show abnormally large fluctuations. Similar behavior is reported for Ta_2_O_5_ films prepared by reactive sputtering and ion beam/electron beam-assisted deposition, where the film stoichiometry and oxygen-vacancy concentration are highly sensitive to process variations when *Q*_2_ changes from an oxygen-deficient to a fully oxidizing regime. On this basis, the range of 75~80 sccm in the present work is considered a sensitive transition region from oxygen-deficient TaO_x_ (x < 2.5) to near-stoichiometric Ta_2_O_5_, which leads to increased sample-to-sample scattering of n. When *Q*_2_ is further increased above 85 sccm, *Q*_2_ near the substrate is sufficient to stabilize the Ta_2_O_5_ phase. In summary, the variations in *n* of Ta_2_O_5_ films are jointly governed by the ion energy effect, the ion flux effect, and the oxidation stoichiometry. A moderate combination of *V*, *I*, *Q*_2_, and *Q*_2_ promotes surface densification and sufficient oxidation, and thus increases n. In contrast, excessive ion energy or over-bombardment induces resputtering, a loose microstructure, or oxygen deficiency, which degrades the optical performance of the films. These trends are consistent with the classical models for IAD growth of oxide thin films [32,38,39].

**Figure 6:**
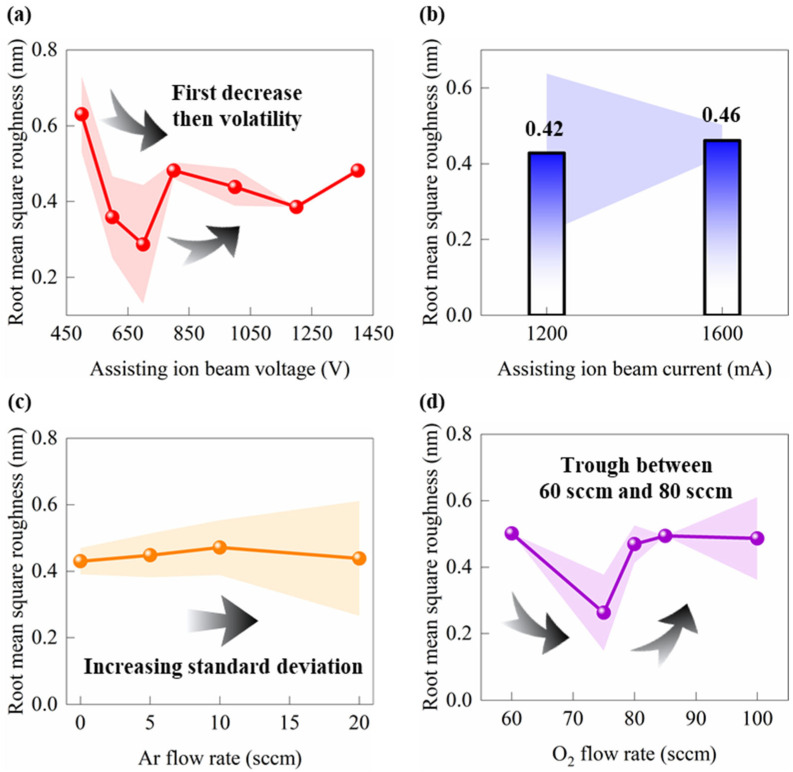
The influence of characteristic parameters on *Rq* of Ta_2_O_5_ optical films. (**a**) Assisting ion source beam voltage—*V* (500~1400 V). (**b**) Assisting ion source beam current—*I* (1200~1600 mA). (**c**) Ar flow rate—*Q*_1_ (0~20 sccm). (**d**) O_2_ flow rate—*Q*_2_ (60~100 sccm). Arrows indicate the general trend of the data; shaded bands represent uncertainty bands.

Figure 6 shows the dependence of *Rq* on four ion source parameters: *V*, *I*, *Q*_1_, and *Q*_2_. In Figure 6a, *Rq* decreases markedly as *V* increases from 450 V to 700 V, reaches a minimum around 700 V, and then increases again when the voltage is further raised to 1000 V. This trend indicates that, in the low and medium voltage range, appropriate incident ion energy promotes surface diffusion and rearrangement of adatoms or clusters. Porosity is closed, and the film becomes denser, which leads to a low *Rq*. When *V* exceeds a certain threshold, high-energy ions enhance resputtering and severe surface etching, so *Rq* increases again [40]. Therefore, there is an optimal voltage range (600~800 V) for minimizing *Rq*. To obtain a low *Rq*, prolonged operation at excessively high *V* should be avoided. Figure 6b shows that *Rq* increases slowly from about 0.38 nm to about 0.42 nm as *I* rises from 1200 mA to 1600 mA. The error band becomes narrower, and the overall variation remains very small. This result indicates that, in the present experimental range, the effect of increasing ion flux on *Rq* is relatively weak. The slight increase in roughness can be attributed to enhanced resputtering, local heating, or stress concentration at high ion flux, which introduces fine surface undulations [40]. Optimization of *I* should therefore be considered together with the beam voltage. At the optimal voltage, the current should be kept at a moderate level to avoid entering a high-flux regime where ion-induced damage dominates. In Figure 6c, when *Q*_1_ increases from 5 sccm to 20 sccm, *Rq* remains around 0.4 nm with a slight decreasing trend and relatively wide error bars. *Q*_1_ affects the discharge characteristics and stability of the ion source. However, in this range, the discharge remains stable, and the surface diffusion of Ta_2_O_5_ is mainly governed by ion energy. A larger gas flow may broaden the ion beam, but a flow of 5~20 sccm likely still lies within an effective bombardment regime. As a result, the overall impact of *Q*_1_ on *Rq* is limited. As shown in Figure 6d, when *Q*_2_ increases from 60 sccm to 100 sccm, *Rq* exhibits a minimum around 75 sccm, a pronounced peak at 80 sccm, and then decreases again in the range of 90~100 sccm. At low oxygen partial pressure, Ta_2_O_5_ films tend to contain a high concentration of oxygen vacancies and sub-stoichiometric TaO_x_ (x < 2.5). The corresponding reduction in density and possible incorporation of inert gas favor the formation of columnar or porous structures, which increases *Rq* [37]. *Rq* minimum when *Q*_2_ increases from 80 sccm to 90 sccm suggests the existence of an optimal near-stoichiometric and highly dense oxygen flow window, in which absorption loss is minimized and the density of micro-defects is low [36]. When *Q*_2_ is further increased, while the *V* and *I* are kept constant, the energy gained per ion decreases. The effective bombardment of the substrate is weakened, and *Rq* increases again [36]. By combining the trends of the four ion source parameter, it is evident that *V* dominates film densification and resputtering behavior and is the most critical factor for *Rq*. I controls the ion flux, its influence is weaker but may still induce damage at high flux. The effect of *Q*_1_ is limited and mainly related to sustaining a stable discharge. In contrast, *Q*_2_ exhibits an optimal window and is the other key factor responsible for abrupt changes in roughness. Overall, these parameters jointly determine the ion energy flux density and the oxidation environment, thus controling the growth kinetics of Ta_2_O_5_ and the final surface morphology.

**Figure 7:**
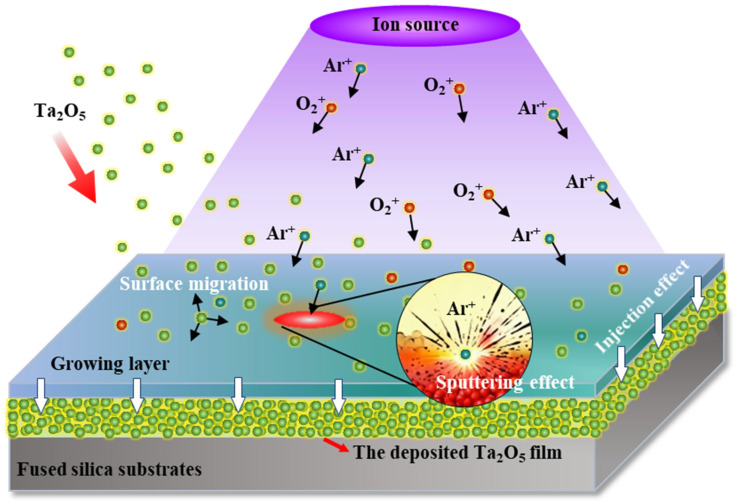
Schematic diagram of the growth mechanism of Ta_2_O_5_ films assisted by ion sources, where surface migration, sputtering effect, and injection effect exist in the growing layer. Arrows indicate the direction of ion and molecule movement; the gray, green, and blue layers represent the fused silica substrate, the deposited Ta_2_O_5_ film, and the growing layer, respectively; the purple region denotes irradiation from the ion source.

The schematic of the IAD-EBE reactive evaporation process for Ta_2_O_5_ films is shown in Figure 7. The Ta_2_O_5_ material is heated by the electron beam and evaporated, and the resulting vapor is transported to the substrate and condenses on the FSS. At the same time, the RF ion source installed in the chamber ionizes the working gas (Ar/O_2_ mixture) and generates Ar^+^ and O_2_^+^ ion beams, which is directed toward the deposition region. In the growing layer, low-energy ions collide with adatoms on the surface. The surface mobility and diffusion length of these adatoms are increased, which promotes cluster rearrangement, pore closure, and interface densification. As a result, *Rq* is reduced and *n* is increased [41]. When the ion energy becomes too high, reflection occurs. High-energy ions knock surface atoms out of the film and generate point defects or stress concentrations near the surface. The deposition rate is reduced, the surface becomes rougher, and the stoichiometry deviates from the nominal value [42]. At the same time, part of the incident ions is trapped within a few nanometers below the growing layer, giving rise to an ion implantation effect, which causes local structural damage and impurity incorporation in the Ta_2_O_5_ layer [40,43]. In the mixed plasma, Ar^+^ mainly acts as an inert momentum carrier. It enhances surface diffusion, increases film density and refractive index. In contrast, O_2_^+^ provides a strong chemical contribution: reactive oxygen ions can oxidize sub-stoichiometric TaO_x_, refill oxygen vacancies, and therefore control the stoichiometry and micro-defect structure of Ta_2_O_5_, which in turn has a pronounced impact on n [36,44]. Overall, enhancement of surface diffusion, resputtering, and the ion implantation effect act cooperatively under the mixed Ar^+^/O_2_^+^ ion beam. These coupled processes provide the basis for obtaining highly dense, low-defect Ta_2_O_5_ films by IAD-EBE.

### 3.2. PI-BO-Based Prediction and Experimental Validation of Rq and n for Ta_2_O_5_ Films

**Figure 8:**
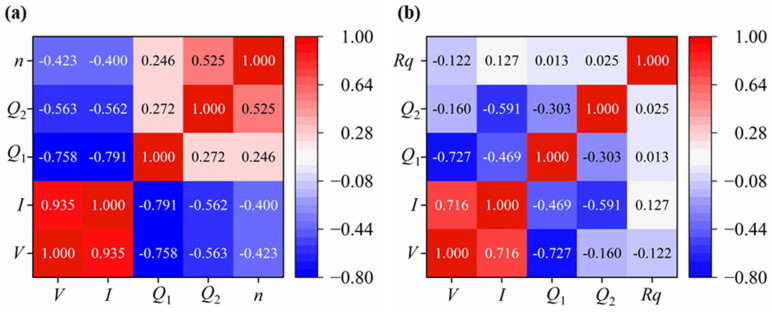
Pearson correlation heatmap of input–output parameters. (**a**) The correlation coefficients of *V*, *I*, *Q*_1_, and *Q*_2_ with *n* show that *Q*_2_ has the strongest positive correlation and *V* has the strongest negative correlation. (**b**) The correlation coefficients of *V*, *I*, *Q*_1_, and *Q*_2_ with *Rq* show that *Q*_2_ has the strongest positive correlation and *I* has the strongest negative correlation.

Figure 8 shows the Pearson correlation coefficient heatmap between *V*, *I*, *Q*_1_, and *Q*_2_ of the Ta_2_O_5_ films and *n*, *Rq*, respectively. The *n* is moderately and negatively correlated with *V* and *I* (*r_n_*_,*V*_ = −0.423, *r_n_*_,*I*_ = −0.4). In contrast, it is more strongly and positively correlated with *Q*_2_ (*r_n_*_,*Q*2_ = 0.525) and only weakly and positively correlated with the *Q*_1_ (*r_n_*_,*Q*1_ = 0.246). The root-mean-square roughness *Rq* shows only weak correlation with any single ion source parameter (|*r*| ≤ 0.13), which is consistent with the results discussed in Section 3.1.

In the PI-BO model proposed in this study, the setting of the physical constraint layer is based on the data statistical laws such as the Ta_2_O_5_ film deposition mechanism analysis and Pearson correlation coefficient analysis in Section 3.1. The specific physical laws are shown in Table 2.

**Figure 9:**
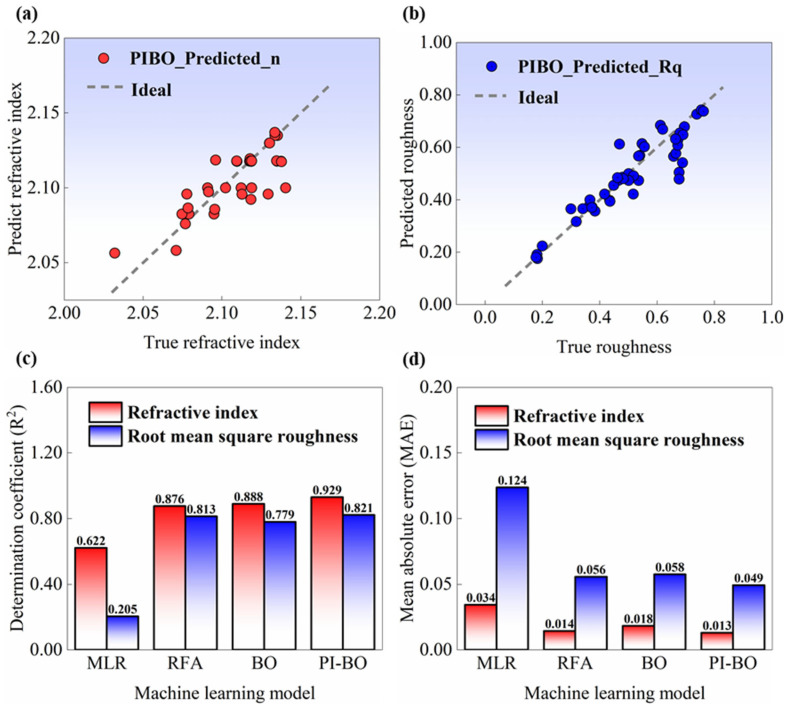
Predictive performance of the PI-BO model and comparative evaluation of machine learning models for Ta_2_O_5_ film properties. (**a**) Comparison between PI-BO model-predicted *n* and true *n* of Ta_2_O_5_ films; the dashed line represents the ideal prediction. (**b**) Comparison between PI-BO model-predicted *Rq* and true *Rq* of Ta_2_O_5_ films. (**c**) R^2^ of different machine learning models (ML, RF, BO, PI-BO) for predicting the refractive index and root-mean-square roughness of Ta_2_O_5_ films. (**d**) MAE of different machine learning models (ML, RF, BO, PI-BO) for predicting *n* and *Rq* of Ta_2_O_5_ films.

The training results of the PI-BO model and performance comparisons across different models are presented in Figure 9. In Figure 9a, the scatter plot of predicted versus actual *n* derived from the PI-BO model is illustrated. Data points are closely distributed around the ideal line, covering an actual *n* range of approximately 2.00 to 2.20. The vast majority of points fall within the band of ±0.05 relative to the ideal line, demonstrating high accuracy and low bias of the model. In Figure 9b, the prediction of *Rq* by the PI-BO model is shown. All data points lie close to the ideal line across the actual *Rq* range of 0.00 to 0.75 nm, with deviations mainly concentrated in the high *Rq* region.

ML models commonly applied to small-sample scenarios include multivariate linear regression (MLR), random forest algorithm (RFA), and standard BO. Figure 9c compares the *R*^2^ of four models for *n* and *Rq* prediction. For *n*, R^2^ value of the MLR model is 0.622, that of the RFA model is 0.876, that of the BO model is 0.888, and the PI-BO model achieves the highest value of 0.929. For *Rq*, the MLR model yields the lowest *R*^2^ of 0.205, followed by the RFA model (0.813), the BO model (0.779), and the PI-BO model (0.821). Superior performance of the PI-BO model over all other models is observed in both metrics, which indicates that it can better capture the nonlinear relationships between input parameters and output properties. Figure 9d displays the mean absolute error (MAE) values of the models. For refractive index, the MAE of the MLR model is 0.034, that of the RFA model is 0.018, that of the BO model is 0.014, and the PI-BO model attains the lowest MAE of 0.013. For roughness, the MLR model has the highest MAE of 0.124, while the RFA model and BO model have MAEs of 0.056 and 0.058, respectively, and the PI-BO model achieves an MAE of 0.049. The low MAE of the PI-BO model highlights its advantages in quantifying prediction errors, especially in the presence of noisy data or limited sample sizes.

Among the four models, the PI-BO model exhibits the highest *R*^2^ and the lowest MAE, which verifies its superior predictive accuracy and fitting performance. The BO and PI-BO models can form a set of ablation experiments. By comparing their prediction results, a robust mapping relationship of the PI-BO model is confirmed. The superiority of the PI-BO model stems from the integration of physical knowledge into the BO process: while the standard BO model only relies on the GP surrogate model to optimize the acquisition function, the PI-BO model constrains the surrogate function via physical prior rules, which enhances the exploration–exploitation balance. In deposition processes, this mechanism helps handle multimodal parameter spaces, thus achieving higher predictive accuracy and efficiency.

### 3.3. Sensitivity Analysis and Optimization of Process Parameters for Deposition Quality

**Figure 10:**
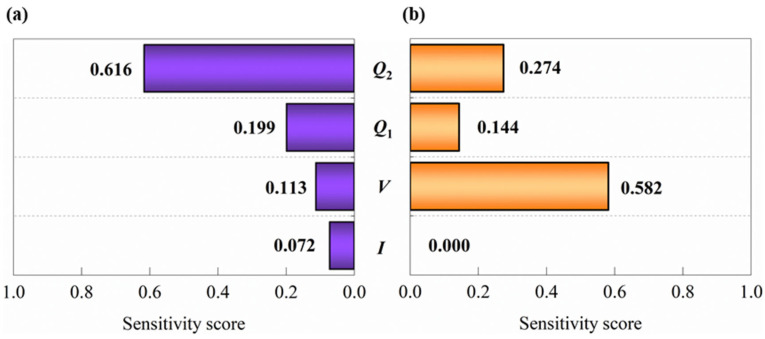
The characteristic importance of the process parameters of Ta_2_O_5_ films was analyzed by using the sensitivity scoring method. (**a**) The contribution of the characteristic parameters of the ion source to *n* is that *Q*_1_ makes the greatest contribution. (**b**) The contribution of the characteristic parameters of the ion source to *Rq* is that *V* contributes the most.

A sensitivity analysis of the assist ion source parameters is carried out based on the PI-BO model to evaluate their relative importance for *n* and *Rq* of Ta_2_O_5_ films deposited by IAD-EBE, as shown in Figure 10. The ion source parameters include *V*, *I*, *Q*_1_, and *Q*_2_. The sensitivity score ranges from 0 to 1 and represents the contribution of each parameter to the target property. The scores are obtained by permutation importance.

Figure 10a shows that the *Q*_2_ is the most important parameter for *n*, with a sensitivity score of 0.616. It is followed by *Q*_2_ (0.199), *V* (0.113), and *I* (0.072). This result indicates that the gas flow parameters dominate the variation in *n* and highlight the primary role of film stoichiometry and oxidation. The dominant role of the *Q*_2_ arises from its direct control of the oxidation state and the Ta:O stoichiometry. In IAD-EBE, O_2_ acts as a reactive gas and promotes ion-activated oxygen incorporation. The formation of sub-oxides (TaO_x_, x < 2.5) is suppressed, the film density is increased, and *n* is raised. *Q*_1_ is secondary and mainly influences the plasma density and ion flux. In this way, it affects surface diffusion and densification and assists the reactive role of O_2_. The lower importance of the *V* and *I* suggests that ion energy and ion flux act mainly as auxiliary factors for *n* control.

Figure 10b shows that *V* is the dominant parameter governing *Rq*, with a sensitivity score of 0.582. It is followed by *Q*_2_ (0.274), *Q*_1_ (0.144), and *I* (0.000). This trend is in sharp contrast to that of *n* and indicates that physical bombardment parameters are prioritized in the control of surface morphology. The high sensitivity of *V* is attributed to the fact that the ion kinetic energy directly drives the surface smoothing process. *Q*_2_ plays a secondary role by modifying the film stoichiometry. The low importance of *Q*_1_ is associated with its role in maintaining a stable ion flux and promoting uniform bombardment. The very low sensitivity of *I* suggests that its variation in the range of 1200~1600 mA has no significant effect on the change in *Rq*.

**Figure 11:**
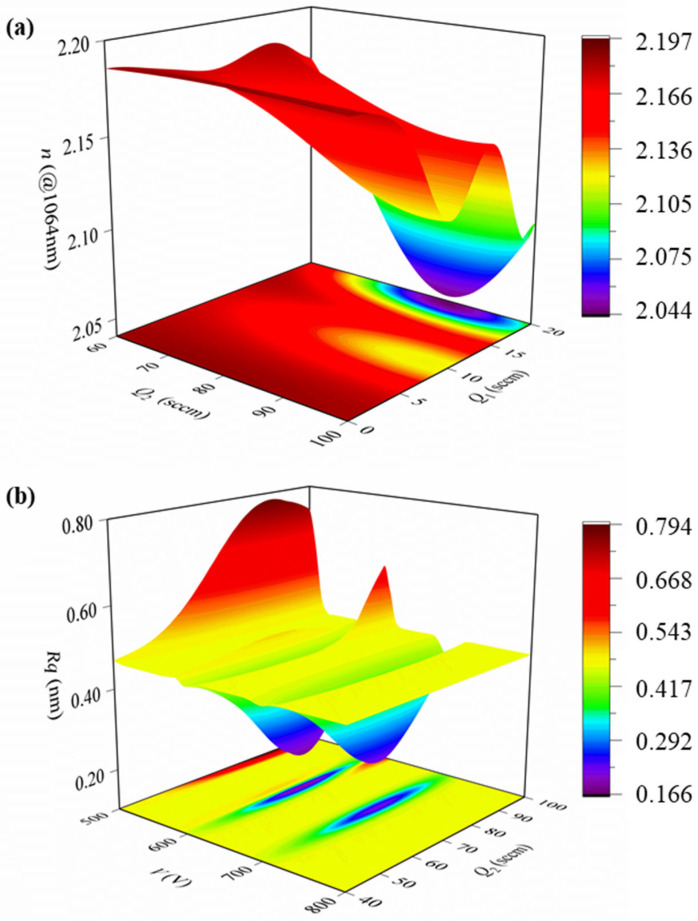
Process contour maps generated by sensitive parameters of the PI-BO model. (**a**) Process contour map of oxygen flow rate and argon flow rate on refractive index of optical film. (**b**) Process contour map of ion source beam pressure and oxygen flow rate on optical film roughness.

Figure 11 presents contour plots of the key process parameters versus *n* and *Rq* of the Ta_2_O_5_ films, as predicted by the PI-BO model. These plots allow the coupling between parameters and their optimization ranges to be visualized directly. In Figure 11a, the overall trend for *n* is as follows: At low *Q*_1_, *n* increases slightly with increasing *Q*_2_ and then tends to saturate, whereas a pronounced valley in n appears at high *Q*_1_. A region with moderate *Q*_2_ (70~80 sccm) and low *Q*_1_ (0 sccm~5 sccm) is favorable for increasing the film density and achieving a higher refractive index. Figure 11b shows a valley of minimum *Rq* at intermediate *V* (600~700 V) combined with intermediate *Q*_2_ (60~80 sccm). In contrast, low or excessively high voltages and too low or too high *Q*_2_ all lead to a marked increase in *Rq*. A combined analysis of the contour distributions for *n* and *Rq* indicates that the optimal process windows for these two properties do not fully overlap. Preferred *V* (600~700 V), and *Q*_2_ (70~80 sccm) together define a relatively narrow optimal range characterized by a high *n* (>2.15) and low *Rq* (0.2~0.3 nm), which provides clear process guidelines for the rapid optimization of Ta_2_O_5_ laser coating performance in engineering practice by adjusting *V* and *Q*_2_.

## 4. Conclusions

This study addresses the challenges associated with fabricating Ta_2_O_5_ films via IAD-EBE, including high experimental costs and unclear coupling effects of IAD parameters on deposition quality. In this work, a small-sample dataset is developed by IAD-EBE experiments and characterization of deposition quality. Utilizing a PI-BO method, accurate prediction of *Rq* and *n* for Ta_2_O_5_ films is achieved. Meanwhile, the sensitivity weight factors and preferred ranges of critical IAD parameters influencing deposition quality are obtained. The main conclusions are summarized as follows:

1. The regulating effects of ion source parameters (*V*, *I*, *Q*_1_ and *Q*_2_) on *n* and *Rq* of Ta_2_O_5_ films are clarified. As *V* increases, the overall trend of *n* and *Rq* shows a fluctuating decline. The increase in *Q*_2_ is conducive to enhancing the oxidation degree of the film, inhibiting the thermal decomposition of Ta_2_O_5_ and oxygen vacancies under high temperature and ion bombardment, while *I* and *Q*_1_ exert secondary influences via flux-induced damage and discharge stability.
2. The proposed method is grounded in the analysis of deposition mechanisms and Bayesian optimization theory. An approach for predicting the deposition quality of Ta_2_O_5_ films and characterizing the features of process parameters is developed, specifically designed for small-sample datasets. The validation results of the PI-BO method show that the *R*^2^/MAE of the prediction models for *n* and *Rq* reach 0.9273/0.0133 and 0.8214/0.0492, respectively. The PI-BO model has high robustness and strong generalization capability.
3. For *n*/*Rq*, the weight factors of *V*, *I*, *Q*_1_, and *Q*_2_ are 0.616/0.274, 0.199/0.144, 0.113/0.582, and 0.072/0.000, respectively. *V* and *Q*_2_ are identified as the dominant factors for regulating the deposition quality of Ta_2_O_5_ films. The preferred ranges for *V* and *Q*_2_ are determined to be 600~700 V and 70~80 sccm, respectively. Within the above ranges, Ta_2_O_5_ films exhibit high refractive index (*n* > 2.15) and low surface roughness (*Rq*: 0.2~3 nm).

In the present work, refractive index and surface roughness were selected as representative film-quality metrics due to their non-destructive measurability and strong correlation with densification behavior. While density was not explicitly included as a target parameter in the machine learning framework, it serves as an important underlying factor governing the observed trends. Incorporating direct density measurements in future studies would further strengthen the structure–property relationships and enhance the interpretability of data-driven optimization models.

## Figures and Tables

**Table 1:** Deposition process parameters of Ta_2_O_5_ film on the fused silica substrates.

Process Parameters	Symbol	Unit	Value Range
Assisting ion source beam voltage	*V*	V	500~1400
Assisting ion source beam current	*I*	mA	1200~1600
Argon (Ar) flow rate	*Q*_1_	sccm	0~20
Oxygen (O_2_) flow rate	*Q*_2_	sccm	60~100

**Table 2:** Physical constraint rules for refractive index and roughness optimization of Ta_2_O_5_ films.

Constraint Rule	Output	Parameter Condition	Weight
Voltage–current synergy rule	*Rq*	*V* ∈ [900, 1000] V & *I* ∈ [1280, 1320] mA	0.20
	*n*	*V* ∈ [800, 1200] V & *I* ∈ [1300, 1500] mA	0.22
Power optimization rule	*Rq*	*P* = *V* × *I*/1000 ∈ [900, 1100] W	0.26
	*n*	*P* = Voltage × Current/1000 ∈ [1000, 1500] W	0.22
Ar flow rate control rule	*Rq*	*Q*_1_ ∈ [7, 9] sccm	0.30
	*n*	*Q*_1_ = 0 sccm	0.18
O_2_ flow rate priority rule	*Rq*	*Q*_2_ ∈ [77, 80] sccm	0.18
	*n*	*Q*_2_ > 90 sccm	0.25
*Q*_2_/*Q*_1_ ratio rule	*Rq*	*Q*_2_/*Q*_1_ ∈ [8, 12] (*Q*_1_ ≥ 5 sccm)	0.15
	*n*	*Q*_2_/*Q*_1_ > 10	0.15

## Data Availability

The data presented in this study are available on request from the corresponding author.

## Abbreviations

The following abbreviations are used in this manuscript:

Ta_2_O_5_	Tantalum pentoxide
*n*	Refractive index
IAD-EBE	Ion-assisted electron beam evaporation
*V*	Assisting ion source beam voltage
ML	Machine learning
TFNNs	Thin-film neural networks
*I*	Assisting ion source beam current
*Rq*	Root-mean-square surface roughness
PI-BO	Physics-informed Bayesian optimization
FSS	Fused silica substrate
RF	Radio frequency
QCM	Quartz crystal microbalance
*Q*_1_	Argon flow rate
*Q*_2_	Oxygen flow rate
AFM	Atomic force microscopy
GP	Gaussian process
MLR	Multivariate linear regression
RFA	Random forest algorithm
MAE	Mean absolute error
